# Effect of drought acclimation on sugar metabolism in millet

**DOI:** 10.1007/s00709-024-01976-5

**Published:** 2024-08-05

**Authors:** Joseph N. Amoah, Monica Ode Adu-Gyamfi

**Affiliations:** 1 School of Life and Environmental Sciences, University of Sydney, 380 Werombi Road, Brownlow Hill, Camden, NSW 2570 Australia; 2 Plant Biotechnology Department, CSIR – Crop Research Institute, Kumasi, Ghana; 3 King Mongkut’s University of Technology Thonburi, Bangkok, Thailand

**Keywords:** Drought acclimation, Osmotic adjustment, Millet, Total sugar transport, Metabolism, Transcriptional regulation

## Abstract

Drought stress triggers sugar accumulation in plants, providing energy and aiding in protection against oxidative damage. Plant hardening under mild stress conditions has been shown to enhance plant resistance to severe stress conditions. While sugar accumulation and metabolism under drought stress have been well-documented in crop plants, the effect of drought acclimation treatment on sugar accumulation and metabolism has not yet been explored. In this study, we investigated the impact of drought stress acclimation on sugar accumulation and metabolism in the leaves and root tissues of two commonly cultivated foxtail millet (*Setaria italica* L.) genotypes, 'PI 689680' and 'PI 662292'. Quantification of total sugars (soluble sugar, fructose, glucose, and sucrose), their related enzymes (SPS, SuSy, NI, and AI), and the regulation of their related transcripts (*SiSPS1*, *SiSuSy1*, *SiSWEET6*, *SiA-INV*, and *SiC-INV*) revealed that drought-acclimated (DA) plants exhibited levels of these indicators comparable to those of control plants. However, under subsequent drought stress conditions, both the leaves and roots of non-acclimated plants accumulated higher levels of total sugars, displayed increased activity of sugar metabolism enzymes, and showed elevated expression of sugar metabolism-related transcripts compared to drought-acclimated plants. Thus, acclimation-induced restriction of sugar accumulation, transport, and metabolism could be one of the metabolic processes contributing to enhanced drought tolerance in millet. This study advocates for the use of acclimation as an effective strategy to mitigate the negative impacts of drought-induced metabolic disturbances in millet, thereby enhancing global food security and promoting sustainable agricultural systems.

**Supplementary information:**

The online version contains supplementary material available at 10.1007/s00709-024-01976-5.

## Introduction

The growth, development, and yield of agriculturally important crops have been significantly impacted by climate change, putting the world's food supply and distribution system in danger (Arora 2019; Malhi et al. 2021). Drought stress, a significant contributor to climate change, adversely affects crop development and yield due to a variety of physical damages, physio-biochemical changes, and molecular alterations that affect various plant functions. These effects include inhibiting photosynthesis, increasing oxidative stress, altering metabolism, and ultimately leading to plant death (Amoah et al. 2019; Kirova et al. 2021; Ru et al. 2023). Considering that the growth and development of millet and other crops are highly dependent on the magnitude and duration of the stress, these plants have evolved various defense and adaptation strategies such as stomata closure, cellular adaptations, changes in carbon fixation rates, hormonal regulation, increased sugar accumulation (osmoregulation), and regulation of stress-expressed signaling genes (Bandurska 2022; Ullah et al. 2017).

The exposure of plants to initial drought stress has been demonstrated to improve tolerance to subsequent stress of greater or similar intensity, a mechanism called hardening or acclimation (Selote et al. 2004; Wilson and Franklin 2002; Woods and Harrison 2002). It is a feasible approach that involves the application of partial water to seedlings to prepare them for drought and other environmental conditions (Thomas 2009). The mechanism has been applied in different crops under different abiotic stress conditions (Ghanbari and Kordi 2019; Kacienė et al. 2017; Khan et al. 2021; Selote and Khanna-Chopra 2010). In our previous study on wheat, we found that drought hardening treatment induced an acclimation response in wheat-rye translocation and non-translocation lines against subsequent drought stress (Amoah et al. 2019; Amoah and Seo 2021). Furthermore, in our recent studies, drought hardening was associated with enhanced polyphenol accumulation, antioxidants activity, as well as starch synthesis and metabolism in foxtail millet (*Setaria italica* L.) genotypes (Amoah et al. 2023, 2024). However, the mechanisms underlying sugar accumulation and metabolism under drought acclimation conditions remain unclear, particularly in millet.

Sucrose is the main carbohydrate transported by plants from mature leaves to roots, and the effectiveness of the phloem transport system, which includes the sypmplastic and apoplastic pathways, is crucial to this mechanism (Ruan 2012). Sucrose unloading in the symplast pathways is achieved by the plasmodesmata, while the transmembrane sucrose transporter proteins in the apoplastic pathways, such as *SWEET* and *SUC* families, also play a role (Jogawat et al. 2021; Yadav et al. 2015). Sucrose secretion and transport mechanisms have been elucidated in plants, such as *Arabidopsis thaliana* (Chen et al. 2012). Subsequently, sucrose is taken up from the companion cells to the sieve cells via the plasmodesma, facilitating transport to the sink organ with the help of *SUC2* (Chen et al. 2012). Furthermore, in plants, sucrose transporters are involved in the re-distribution of sucrose between source and sink during abiotic stress conditions (Durand et al. 2016; Lemoine et al. 2013) However, there is no information about the regulatory functions of sucrose transporters in plants under drought acclimation conditions. Therefore, understanding the dynamic mechanisms driving sucrose transport and assimilation under drought acclimation will provide valuable information for breeders in selecting the best millet genotype in a breeding program aimed at developing drought-tolerant millet genotypes. This is crucial for addressing the global food crisis and ensure environmental sustainability.

The objectives of this study are: (a) To investigate the impact of drought hardening on millet growth, (b) To elucidate the mechanisms underlying drought-induced tolerance in plants exposed to either drought-acclimation or non-acclimation, with a particular focus on sugar accumulation, transport, and metabolism, (c) To examine the regulation of transcripts associated with sugar transport in plant tissue undergoing either drought acclimation or non-acclimation during drought stress. This research supports the idea that drought hardening enhances drought stress tolerance by enhancing sugar accumulation, transport, and metabolism, aspects that are not yet fully understood in plants. The findings of this study will provide a basis for future investigations into the practical application of hardening to improve crop productivity under drought conditions.

## Materials and methods

### Plant materials, growth condition, and stress treatment

**Fig.1:**
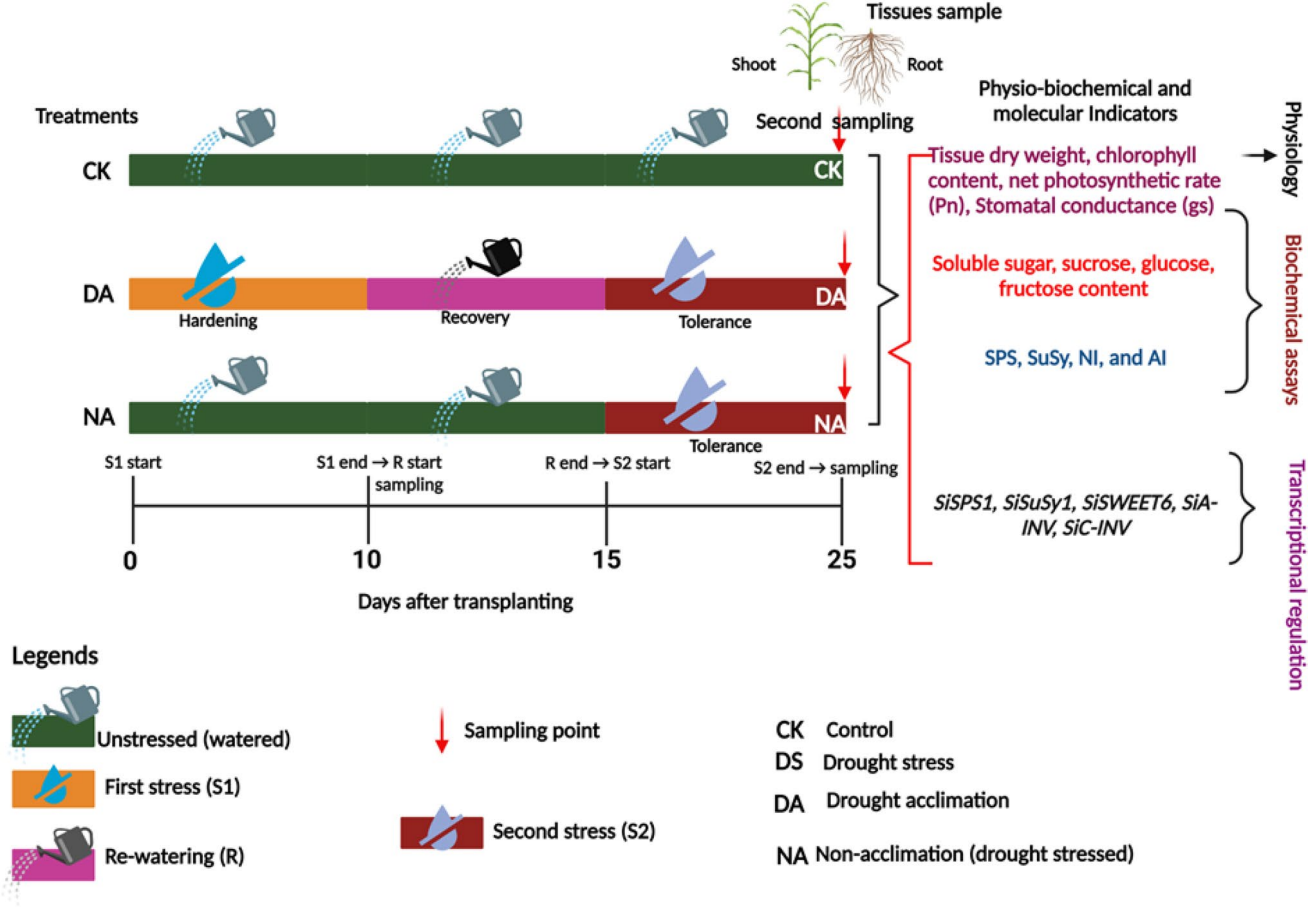
The diagram illustrates the experimental framework. Seedlings of millet genotypes ‘PI 662292’ and ‘PI 689680’ were divided into three groups: the control group (CK, unstressed), the drought acclimation group (DA; exposed to two stress episodes, S1 followed by S2, with a recovery ® period), and the non-acclimation group (NA, subjected to a single stress episode, S2, synchronized with DA without any recovery). The hardening stress (S1) began at 0 days after transplanting the seedlings (DAT), continued until 10 DAT, and was followed by the sampling of shoot and root tissues for both CK and drought stress (DS) plants. Afterward, the seedlings were watered for 5 days to facilitate recovery (R) and then subjected to another stress episode (S2) for 10 days (from 15 to 25 DAT). Sampling was conducted at 25 DAT for CK, DA, and NA plants. CK, DS, DA, NA, and DAT represent control, drought stress or hardening, drought acclimation, non-acclimation, and days after seedling transfer, respectively

Seeds of the commonly cultivated millet genotypes, 'PI 662292' and 'PI 689680', were obtained from the United States Department of Agriculture (USDA). These millet genotypes were chosen based on our previous studies, which revealed differential polyphenol and starch accumulation under drought stress conditions (Amoah et al. 2023, 2024). The seeds were surface sterilized in 70% ethanol for 2 min, treated with 8% sodium hypochlorite for 5 min, and then washed five times with sterile distilled water and germinated on moist paper in a petri dish kept in the dark. Subsequently, 3-day-old seedlings were transferred and grown in pots (10 × 10 × 8 cm) containing soil (sunshine mix #2) in a glasshouse under a photocycle of 16:8 h (day: night), 25–22 °C (day-night), 80% relative humidity (RH), and active photosynthetic radiation at 600 mol m^−2^ s^−1^ with supplemental LED lights for 10 d before the drought stress treatments were initiated. The conditions in the glasshouse were monitored using a Campbell data logger device (Campbell Computer, Bourne, MA, USA). The seedlings were at the fully expanded 3rd leaf stage according to Zadok’s scale #13 (Zadoks et al. 1974) after 10 d, and uniformly grown seedlings were separated into three groups: control (CK), drought acclimation (DA), and non-acclimation (NA) (Khanna-Chopra and Selote 2007). Control (CK) plants were watered daily with half strength Hoagland nutrient solution. Drought acclimation (DA) plants were exposed to a first stress (S1) for 10 d, rewatered for 5 d, and then subjected to a second stress (S2) for 10 d. The non-acclimation (NA) plants were exposed to a single stress episode, synchronous with a second stress (S2) in the DA plants. Plant tissue (shoot and root) sampling was done 10 days after transplanting (DAT) for S1 and at 25 DAT for S2 (Fig. 1). During sampling, shoot and root tissues were harvested in two different sets; the first sets were oven-dried for 48 h, and the dry weights were estimated with chemical balance. The second set was immediately frozen in liquid N_2_ and stored in the freezer (-80 °C) for use in various biochemical assays and transcript expression analysis.

### Net photosynthetic rate, biomass, and relative water content

Photosynthetic parameters were measured on the third fully expanded leaf of each plant after 15 days (S2) for control (CK), drought acclimation (DA), and non-acclimation (NA) plants. The measurements were taken at 9:00 AM and 11:00 AM using an LI-6800 portable photosynthetic system (LI-COR Inc., Lincoln, NE, USA). The light-saturation point was set to µmol (photon) m^−2^ s^−1^ to measure the net photosynthetic rate and stomatal conductance using the methods reported by Du et al. (2020a) and Li et al. (2017). The ambient temperature of each measured millet leaf was set to 30 °C. The CO_2_ concentration was 400 µmol (CO_2_) mol^−1^, the relative humidity was 60%-65%, and the airflow was 500 µmol. For tissue relative water contents (RWC), the leaves and roots of CK, DA, and NA-treated plants were sampled. Fresh weights were immediately taken, and the samples were incubated in distilled water for 4 h with vigorous shaking. The turgid weights (TW) were measured after 4 h, and the tissue samples (leaf and roots) were oven-dried at 60 °C for 48 h, after which the dry weights (DW) were recorded. Tissue RWC was estimated using the equation.

1 $$\text{RWC}(\text{\%})=\frac{(FW-DW)}{TW-DW}\times 100$$

### Soluble sugar, sucrose, fructose, and glucose content

To determine the total soluble sugar and sucrose, the leaves, and roots of control (CK), drought acclimation (DA), and non-acclimation (NA) plants were sampled, snap-frozen, and extraction was done according to previous methods by Du et al. (2020b) with minor modifications. Briefly, 100 mg of ground samples were homogenized in 1 mL of 80% (v/v) ethanol, and the mixture was heated at 80 °C for 30 min. The mixture was allowed to cool for 5 min and centrifuged at 12,000 × *g* for 10 min. The supernatants were separated, and soluble sugar and sucrose contents were determined by recording absorbance at 620 nm and 480 nm wavelengths, respectively using a spectrophotometer (UV-2550, Shimadzu, Japan).

Fructose content was determined using hydroxyphenol colorimetry as described by Dong et al. (2023). To achieve this, 1 mL of extract, 1 mL of 0.1% hydroquinone, and 3.5 mL of 30% HCL were combined in a test tube, thoroughly mixed, and heated at 80 °C for 10 min in a water bath. Subsequently, the absorbance at a wavelength of 480 nm was measured using a spectrophotometer after the solution had cooled and the blank had been adjusted to 0. The recorded absorbance value was then used to calculate the corresponding sugar content utilizing a standard curve.

To determine the glucose content, anthrone colorimetry according to Dong et al. (2023) was employed. A mixture of 1 mL of supernatant and 5 mL of anthrone dilute sulfuric acid reagent was boiled for 10 min. Similarly, a blank was prepared using 1 mL of distilled water instead of supernatant. After cooling the solution, the absorbance was measured at a wavelength of 620 nm using a spectrophotometer, and the blank was adjusted to 0.

### Sucrose phosphate synthase (SPS), sucrose synthase (SuSy), alkaline, and soluble acid invertase activity

A 0.1 g of frozen tissue samples were homogenized in an extraction buffer containing 50 mM Tris–HCl (pH 7.5), 1 mM EDTA, 1 mM MgCl_2_, 12.5% (v/v) glycerine, 10% polyvinylpyrrolidone (PVP), and 10 mM mercaptoethanol to ascertain the activities of sucrose metabolism-related enzymes. The SPS and SuSy activities were measured using the supernatant according to the methods described by (Liu et al. 2013). Briefly, 200 μL of supernatant was mixed with reaction buffer containing 200 mM Tris–HCl (pH 7.0), 40 mM MgCl_2_, 12 mM UDPglucose, 40 mM fructose-6-P, and 200 μL extract. Another reaction buffer containing 12 mM UDP, 40 mM sucrose, 200 mM Tris–HCl (pH 7.0), and 40 mM MgCl_2_ was also prepared. The mixture was incubated at 30 °C for 30 min and terminated using 100 μL 2 mol L^−1^ of NaOH. The mixture was then heated at 100 °C for 10 min to destroy untreated hexose and hexose phosphates, cooled to room temperature, and mixed with 1 mL of 0.1% (w/v) resorcin in 95% (v/v) ethanol and 3.5 mL of 30% (w/v) HCl. The solution was incubated for 10 min at 80 °C. Sucrose content in the SPS reaction and fructose content in the SuSy reaction were calculated using a standard curve measured at A480 nm and A540 nm wavelengths, respectively.

The activities of neutral/alkaline invertase (NI) and soluble acid invertase (AI) were measured by incubating 100 µL of extract with 200 µL of 1 M sucrose and either 2.2 mL of 100 mM sodium acetate–acetic acid (pH 7.5) (for alkaline invertase) or 2.2 mL of 200 mM acetic acid–NaOH (pH 5.0) (for acid invertase) for 30 min at 30 °C. The reaction was halted by adding 1 mL of 3,5-dinitrosalicylic acid (DNS) and boiling for 5 min. Glucose contents in AI and NI reactions were then measured spectrophotometrically at A540 nm, following the method described by Liu et al. (2013)

### RNA extraction, cDNA synthesis, and qPCR expression analysis

Total RNA was extracted from leaf and root samples using the Trizol reagent (Invitrogen, Carlsbad, CA, USA) and RNase-free DNase (Promega, Madison, WI, USA) following the manufacturer’s instructions. RNA purity was assessed using a nanophotometer (Implen, Inc., Westlake Village, CA, USA), and the integrity of total RNA was confirmed by ethidium bromide staining analysis through agarose gel electrophoresis. DNA-free total RNA was reverse transcribed into first-strand cDNA using a Power cDNA synthesis kit (Intron Biotechnology Inc., South Korea). Quantitative real-time PCR (qPCR) was conducted using a CFX96 Real-Time system (Bio-Rad, Richmond, CA, USA) with SYBR green fluorescence dye (Bio-Rad, Richmond, CA, USA), and the results were analysed using the ^ΔΔ^CT method. Gene-specific primers (refer to Table S1) for qPCR were utilized to assess their activity under progressive drought stress and acclimation conditions. The thermal cycling conditions included an initial denaturation step at 95 °C for 5 min followed by 40 cycles of 95 °C for 15 s, 55 °C for 15 s, and 72 °C for 30 s. All experiments were performed with three biological replicates, and the relative transcript levels were normalized using *SiActin*, *Situbulin*, and *Siubiquitin* as internal controls.

### Data analysis

Data were analyzed with the R Statistical Software (v4.3.2). Two-way analysis of variance (ANOVA) was done with genotype as the fixed effect and treatments (control, drought acclimation, and non-acclimation) as random factors. The differences between means were assessed using Tukey's multiple range test (*P* < 0.05), and the results are indicated by different letters above the bars. The correlations between physio-biochemical and molecular indicators of millet shoot and roots were determined with Pearson's correlation matrix, using the R Statistical Software (v4.3.2). Heat maps were generated with TBTools Software (v1.108) (Chen et al. 2020). The principal component analysis (PCA) was done by using the R Statistical Software (v4.3.2). Each result was summarized by the mean ± standard error (SE) of three independent experiments. Bar graphs were made with the GraphPad Prism software v9.51 (733) and heat maps were made using TBTools Software (v1.108) (Chen et al. 2020).

## Results

### Changes in physio-biochemical indicators and transcriptional regulation in millet under drought (hardening) conditions

The initial drought treatment (S1) significantly (p ≤ 0.05) decreased tissue dry weight, chlorophyll content, and leaf gas exchange parameters in the drought stressed (DS) plants of millet genotypes compared to those of control (CK) plants. However, the decrease in these physiological indicators was more pronounced in the DS plants of 'PI 689680' than in those of 'PI 662292' (Table 1). For instance, a significant 34%, 26%, 59%, and 52% decrease in shoot biomass, leaf water content, net photosynthetic rate, chlorophyll content, and stomatal conductance, respectively, was observed in DS plants of 'PI 689680', compared to a 22% decrease in shoot biomass, 11% decrease in relative water content (RWC), 13% decrease in net photosynthetic rate, and a 7% decrease in chlorophyll content in the leaves of 'PI 662292' (Table S2). Similarly, in the root tissues, a significant 68% decrease in biomass, 52% decrease in RWC, and a 35% increase in the root-to-shoot (R/S) ratio were observed in 'PI 689680', while these indicators decreased by 19%, 21%, and increased by 3% in 'PI 662292' (Table S2).

**Table 1:** Changes in physio-biochemical indicators in millet after initial drought (S1) treatment

Traits/ (plants)	PI 662292		PI 689680	
	CK	DS	CK	DS
**Shoot (g/DW)** RWC (%) Pn [µmol (CO_2_) m^−2^ s^−1^] Chlorophyll (µmol g/FW) gs (µmol m^−2^ s-1) Soluble sugar (µmol g/FW) Sucrose (µmol g/FW) Fructose (µmol g/FW) Glucose (µmol g/FW) SPS activity (µmol g/FW) SuSy activity (µmol g/FW) NI activity (µmol g/FW) AI activity (µmol g/FW)	1.10 ± 0.08a 85.80 ± 2.09a 13.27 ± 1.06a 88.70 ± 0.91a 0.66 ± 0.05a 20.36 ± 2.3b 51.53 ± 6.24b 10.01 ± 1.08b 1.86 ± 0.37b 9.33 ± 1.85b 20.61 ± 2.49b 11.16 ± 0.49b 7.93 ± 0.52b	0.91 ± 0.10a 77.33 ± 3.90b 11.70 ± 0.59a 82.59 ± 2.15b 0.58 ± 0.03a 29.26 ± 2.47a 88.53 ± 1.98a 14.63 ± 1.23a 2.66 ± 0.33a 26.66 ± 3.33a 35.41 ± 0.79a 23.43 ± 1.57a 18.20 ± 0.32a	1.24 ± 0.03a 92.92 ± 3.02a 14.19 ± 0.35a 93.86 ± 2.57a 0.70 ± 0.01a 21.06 ± 1.15b 64.66 ± 4.98b 10.53 ± 0.57b 1.26 ± 0.17b 6.33 ± 0.88b 25.86 ± 1.99b 12.43 ± 0.97b 9.30 ± 0.75b	0.92 ± 0.02b 73.25 ± 3.48b 8.87 ± 0.34b 61.53 ± 3.49b 0.44 ± 0.02b 35.56 ± 1.13a 85.06 ± 9.89a 15.11 ± 2.20a 3.16 ± 0.34a 31.66 ± 3.40a 34.02 ± 3.95a 17.96 ± 0.88a 15.70 ± 0.92a
Traits/(plants)	PI 662292		PI 689680	
	CK	DS	CK	DS
Root (g/DW) RWC (%) R/S ratio Soluble sugar (µmol g/FW) Sucrose (µmol g/FW) Fructose (µmol g/FW) Glucose (µmol g/FW) SPS activity (µmol g/FW) SuSy activity (µmol g/FW) NI activity (µmol g/FW) AI activity (µmol g/FW)	0.42 ± 0.03a 86.75 ± 1.05a 0.22 ± 0.03a 20.10 ± 1.50b 13.13 ± 4.14b 10.83 ± 0.08b 1.06 ± 0.12b 9.34 ± 2.01b 4.66 ± 1.46b 4.06 ± 0.26b 2.80 ± 0.17b	0.35 ± 0.05a 71.78 ± 3.14b 0.24 ± 0.03a 25.75 ± 1.13a 16.33 ± 1.16a 12.85 ± 0.56a 1.26 ± 0.03a 11.70 ± 0.98a 20.40 ± 4.42 5.33 ± 0.26a 8.10 ± 0.37a	0.64 ± 0.01a 84.84 ± 2.46a 0.25 ± 0.02b 16.66 ± 0.67b 18.66 ± 1.45b 11.36 ± 0.87b 1.26 ± 0.14b 8.42 ± 0.46b 14.40 ± 0.83b 6.33 ± 0.37b 7.03 ± 0.29b	0.38 ± 0.01b 55.74 ± 2.59b 0.39 ± 0.09b 26.33 ± 1.20a 31.50 ± 2.76a 19.66 ± 3.84a 3.23 ± 0.59a 12.09 ± 1.77a 19.11 ± 3.47a 10.03 ± 0.49a 8.90 ± 0.31a

The data shows the mean (± S.E.) of biological triplicates (n = 3) of two independent experiments. Different letters indicate significance at a probability level of (P ≤ 0.05). RWC stands for relative water content; Pn for net photosynthetic rate; gs for stomatal conductance; SPS for sucrose phosphate synthase; SuSy for sucrose synthase; NI for neutral invertase; AI for soluble acid invertase; and FW for fresh weight

Furthermore, initial drought stress (S1) led to a significant (p ≤ 0.05) increase in soluble sugar, sucrose, fructose, and glucose contents in the shoot and root tissues of DS plants from both genotypes. This effect was more pronounced in the leaves than in the roots and was notably higher in the DS plants of 'PI 689680' compared to 'PI 662292', when compared with their respective controls (CK) (Table 1). For example, in 'PI 689680', the leaf soluble sugar, sucrose, fructose, and glucose contents increased significantly (p ≤ 0.05) by 40%, 24%, 30%, and 60%, respectively, while in the roots, they increased by 37%, 41%, 42%, and 61%, respectively. Conversely, in 'PI 662292', there was a 30%, 42%, 32%, and 30% increase in leaf soluble sugar, sucrose, fructose, and glucose contents, respectively, and a 22% increase in soluble sugar, 20% in sucrose, 16% in fructose, and 20% in glucose content in the roots (Table 1 and Table S2).

In parallel to the levels of total soluble sugar, sucrose, fructose, and glucose in the tissues, there was a significant increase (p ≤ 0.05) in the activities of sucrose phosphate synthase (SPS), sucrose synthase (SuSy), and invertases (neutral invertase [NI] and alkaline invertase [AI]) in both genotypes following the S1 treatment (Table 1). The extent of change in these indicators was notably greater in the DS plants of 'PI 689680' compared to those of 'PI 662292' (Table S1 and Table S2). Particularly, in the DS plants of 'PI 662292', there was a significant (p ≤ 0.05) increase of 65% (SPS), 41% (SuSy), 32% (NI), and 46% (AI) in leaf tissues, and 20% (SPS), 27% (SuSy), 24% (NI), and 25% (AI) in roots (Table S2). Likewise, significant increases (p ≤ 0.05) of 80% (SPS), 23% (SuSy), 30% (NI), and 41% (AI) were observed in leaf tissues, and 30% (SPS), 75% (SuSy), 36% (NI), and 41% (AI) in roots of DS plants of 'PI 662292' (Table S2).

**Fig. 2:**
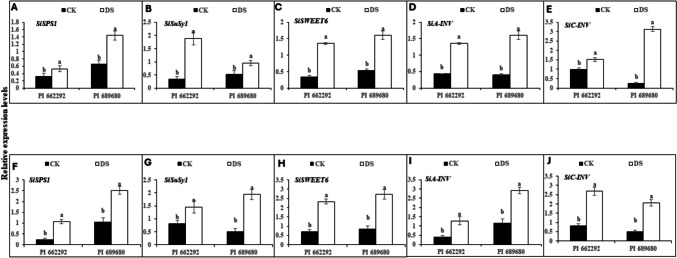
Expression of sugar-related transcripts in the leaves (A-E) and roots (F-J) of millet genotypes after drought hardening (S1) treatment. The relative expression levels of (A) *SiSPS1*, (B) *SiSuSy1*, (C) *SiSWEET6*, (D) *SiA-INV*, and (E) *SiC-INV* in the leaves, and (F) *SiSPS1*, (G) *SiSuSy1*, (H) *SiSWEET6*, (I) *SiA-INV*, and (J) *SiC-INV* in the roots of millet genotypes. CK represents the control group and DS represents the drought stress (hardening) group. The data represent the mean (± S.E.) from biological triplicates. Significant differences are indicated by distinct letters on the error bars, with significance set at a probability level of (p ≤ 0.05)

Corresponding with the levels of soluble sugars (sucrose, fructose, and glucose), the activities of their associated enzymes (SPS, SuSy, NI, and AI), and the expression levels of sugar-metabolism related transcripts (*SiSPS1*, *SiSuSy1*, *SiSWEET6*, *SiA-INV*, and *SiC-INV*) were differentially upregulated in the leaf and root tissues of DS plants from both genotypes following S1 treatment (Fig. 2). For instance, the expression of these transcripts varied from 1.5-fold (*SiC-INV*) to fivefold (*SiSuSy1*) in the leaf and from 1.8-fold (*SiSuSy1*) to 3.4-fold (*SiC-INV*) in the root of ‘PI 662292’. In contrast, the upregulation of these genes increased from 1.8-fold (*SiSuSy1*) to 12.66-fold (*SiC-INV*) in the roots, and from 2.3-fold (*SiSPS1*) to fourfold (*SiC-INV*) in the roots of DS plants of 'PI 689680' (Table S2).

The drought stress (S1) treatment significantly impacted all the studied indicators, except for leaf and root dry weights. Genotype influence was only evident in the expression of *SiSPSS1*, *SiC-INV*, and *SiA-INV* in leaf tissue, and in all indicators in root tissue, except for root dry weight, soluble sugar, fructose, glucose, SuSy, *SiSPS1* expression, and the root-to-shoot (R-S) ratio. Moreover, genotype by treatment (G x T) interaction significantly influenced photosynthetic rate (Pn), chlorophyll content, stomatal conductance (gs), *SiSWEET6* expression, and *SiA-INV* expression in leaf tissues, as well as relative water content (RWC), *SiSuSy1* expression, and *SiSWEET6* expression in root tissues (Table 2).

**Table 2:** Two-way analysis of variance of physiological and molecular indicators under initial drought stress (SI) condition

Traits/(plants)	Variation sources					
	Shoot			Root		
	Genotype (G) (df = 1)	Treatment (T) (df = 1)	G x T (df = 1)	Genotype (G) (df = 1)	Treatment (T) (df = 1)	G x T (df = 1)
Biomass RWC Pn Chlorophyll gs Soluble sugar Sucrose Fructose Glucose SPS SuSy NI-activity AI-activity *SiSPS1* *SiSuSy1* *SiSWEET6* *SiC-INV* *SiA-INV* R/S ratio	0.7576 ns 0.8224 ns 0.3519 ns 0.1481 ns 0.3519 ns 0.2712 ns 0.2539 ns 0.7834 ns 0.2254 ns 0.9024 ns 0.2539 ns 0.1348 ns 0.1946 ns 0.0481* 0.9463 ns 0.2302 ns 0.0094** 0.0012** -	0.0483* 0.0031** 0.0197* 0.033* 0.0197* 0.0147* 0.038* 0.0343* 0.0251* 0.041 ns 0.038* 0.0299* 0.0037** 0.0098** 0.0014** 0.003** 0.003** 0.0028** -	0.1732 ns 0.1111 ns 0.0453* 0.0185* 0.0453* 0.1691 ns 0.3944 ns 0.9927 ns 0.1276 ns 0.6707 ns 0.3944 ns 0.0777 ns 0.1983 ns 0.0603 ns 0.1843 ns 0.0057** 0.1867 ns 0.0005*** -	0.1437 ns 0.0397* - - - 0.3995 ns 0.017* 0.2842 ns 0.0809 ns 0.0042** 0.5117 ns 0.0006*** 0.004** 0.0683 ns 0.0162* 0.0145* 0.0061** 0.0236* 0.3033 ns	0.3372 ns 0.0283* - - - 0.0335* 0.0261* 0.0073** 0.0593 ns 0.0459* 0.0064** 0.003** 0.036* 0.0087** 0.0099** 0.0073** 0.0102* < 0.006** 0.0275*	0.4999 ns 0.0339* - - - 0.1366 ns 0.271 ns 0.3733 ns 0.2397 ns 0.0836 ns 0.4872 ns 0.2226 ns 0.1285 ns 0.0522 ns 0.3758 ns 0.0224* 0.0357* 0.9158 ns 0.4417 ns

^***^, **, * denote significant differences at probability levels of (*P* ≤ 0.001, 0.01, and 0.05), respectively, while 'ns' denotes non-significant differences. RWC represents relative water content; Pn, net photosynthetic rate; gs, stomatal conductance; SPS, sucrose phosphate synthase; SuSy, sucrose synthase; and R/S ratio, Root/shoot ratio. 'G' denotes genotype, 'T' denotes treatment, and 'G' represents genotype

### Changes in physio-biochemical indicators and transcriptional regulation in millet after drought stress (S2) treatment

The second drought stress (S2) treatment, implemented to evaluate the influence of hardening (S1) on millet resulted in a significant (p ≤ 0.05) decrease in tissue biomass, relative water content (RWC), photosynthetic rate (Pn), chlorophyll content, and stomatal conductance, while also increasing the R/S ratio (p ≤ 0.05) in NA plants compared to DA and CK plants (Table 3). The magnitude of change in these indicators was relatively higher in the NA plants than in DA plants of both genotypes (Table S3). In leaf tissue, the significant average decrease in dry weight, RWC, Pn, chlorophyll content, and stomatal conductance for NA plants was 77%, 57%, 94%, 90%, and 89%, respectively, compared to a non-significant 9%, 8%, 7%, 4%, and 5% decrease for DA plants. Additionally, the R/S ratio increased by 15% and 72% for DA and NA plants, respectively. In the roots, a significant 58% decrease in dry weight and a 41% decrease in RWC were observed in NA plants compared to a 16% decrease in dry weight and a 5% decrease in RWC in DA plants (Table S3). From the two-way ANOVA, stress treatment (T) affected only chlorophyll content, root RWC, Pn, and stomatal conductance. Genotype (G) impacted leaf and root RWC, Pn, stomatal conductance, and chlorophyll content, whereas the interaction between genotype and treatment (G x T) impacted only Pn, chlorophyll content, and root RWC (Table 4).

**Table 3:** Changes in physiological indicators in control, drought-acclimated and non-acclimated plants of millet genotypes

Traits/(leaf)	PI 662292			PI 689680		
	CK	DA	NA	CK	DA	NA
Shoot (g/DW) RWC (%) Pn Chl gs	2.82 ± 0.39a 76.79 ± 1.80a 18.63 ± 0.34a 93.19 ± 1.69a 4.65 ± 0.08a	2.58 ± 0.19a 70.72 ± 4.49a 17.26 ± 1.26a 88.83 ± 2.14a 4.44 ± 0.10a	1.56 ± 0.24b 45.69 ± 6.48b 8.87 ± 0.35b 45.39 ± 1.67b 2.27 ± 0.08b	3.11 ± 0.32a 86.24 ± 2.09a 18.33 ± 0.31a 91.66 ± 1.57a 4.58 ± 0.07a	2.66 ± 0.12a 83.25 ± 2.26a 18.25 ± 0.29a 89.58 ± 0.55a 4.48 ± 0.03a	1.85 ± 0.13b 65.90 ± 2.59b 11.75 ± 0.62b 60.74 ± 1.19b 3.04 ± 0.06b
Traits/(Root)	PI 662292			PI 689680		
	CK	DA	NA	CK	DA	NA
Root (g/DW) RWC (%) R/S ratio	0.318 ± 0.03a 84.84 ± 2.43a 0.89 ± 0.09c	0.27 ± 0.03a 81.60 ± 3.39a 1.30 ± 0.23bc	0.20 ± 0.02b 60.72 ± 5.15b 4.25 ± 0.67a	0.33 ± 0.01a 84.86 ± 1.90a 1.42 ± 0.30b	0.29 ± 0.01a 78.28 ± 4.79a 1.68 ± 0.22b	0.21 ± 0.02b 57.95 ± 2.29b 5.05 ± 0.34a

The data shows the mean (± S.E.) of biological triplicates (n = 3) of two independent experiments. Different letters indicate significance at a probability level of (*P* ≤ 0.05). RWC, relative water content; Pn, net photosynthetic rate [(µmol (CO_2_) m^−2^ s^−1^; gs, stomatal conductance; chl, chlorophyll content (µmol g/FW); R/S ratio, root/shoot ratio; CK, control; DS, drought acclimation; and NA, non-acclimation

**Table 4:** Two-way analysis of variance of physiological and molecular indicators under drought stress (S2) condition

Traits/(plants)	**Variation sources**					
	Shoot			Root		
	Genotype (G) (df = 1)	Treatment (T) (df = 2)	G x T (df = 2)	Genotype (G) (df = 1)	Treatment (T) (df = 2)	G x T (df = 2)
Biomass RWC Pn gs Chlorophyll Glucose Soluble sugar Sucrose Fructose SPS SuSy NI activity AI activity *SiSPS1* *SiSuSy1* *SiSWEET6* *SiC-INV* *SiA-INV* R/S ratio	0.3643 ns 0.0633 ns 0.0181* 0.4788 ns 0.0221* 0.0541 ns 0.6321 ns 0.0557 ns 0.5166 ns 0.4853 ns 0.4825 ns 0.007** 0.0369* 0.0056** 0.4049 ns 0.0293* 0.0194* 0.6383 ns -	0.0653 ns 0.0661 ns 0.0018** 0.0244* < 0.0001**** 0.0094* 0.0268* 0.0189* 0.0109* 0.0308** 0.0075** 0.0021** 0.0061** 0.0353* 0.0109* 0.0067* 0.0105* 0.0082* -	0.743 ns 0.3681 ns 0.048* 0.9076 ns 0.0202* 0.2976 ns 0.9418 ns 0.3331 ns 0.0808 ns 0.8072 ns 0.9507 ns 0.8411 ns 0.6332 ns 0.3845 ns 0.0808 ns 0.1256 ns 0.4653 ns 0.0056* -	0.4087 ns 0.0034** - - - 0.0009*** 0.0466* 0.0217* 0.2912 ns 0.0028** 0.0054** 0.022* 0.1674 ns 0.4049 ns 0.1309 ns 0.1036 ns 0.0169* 0.0293* 0.054 ns	0.1155 ns 0.0014** - - - 0.0002*** 0.0008*** 0.0073** 0.0097* 0.0078** 0.0204* 0.0041** 0.001*** 0.0082** 0.0178* 0.0226* 0.0019** 0.031* 0.0264*	0.8303 ns 0.0259* - - - 0.003** 0.0214* 0.019 ns 0.523 ns 0.0243* 0.0139* 0.0229* 0.0461* 0.1538 ns 0.2501 ns 0.2524 ns 0.4653 ns 0.0229* 0.4429 ns

***, **, * denote significant differences at probability levels of (*P* ≤ 0.001, 0.01, and 0.05), respectively, while 'ns' denotes non-significant differences. RWC represents relative water content; Pn, net photosynthetic rate; SC, stomatal conductance; SPS, sucrose phosphate synthase; SuSy, sucrose synthase; and R/S ratio, Root/shoot ratio. 'G' denotes genotype, 'T' denotes treatment, and 'G' represents genotype

**Fig. 3:**
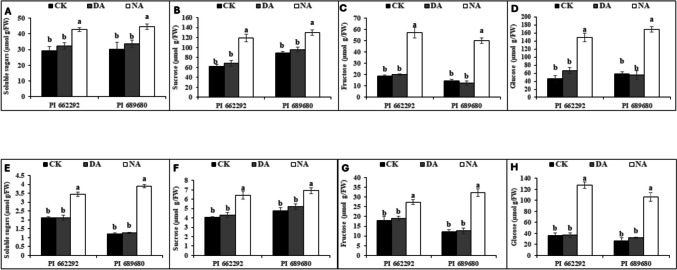
(A) Soluble sugar content, (B) sucrose, (C) fructose, and (D) glucose content in the leaves, and (E) soluble sugar content, (F) sucrose, (G) fructose, and (H) glucose content in the roots of control (CK), drought-acclimated (DA), and non-acclimated (NA) plants after the second stress (S2) treatment. The data represent the mean (± S.E.) from biological triplicates. Significant differences are indicated by distinct letters on the error bars, with significance set at a probability level of (p ≤ 0.05). CK, DA, NA represents control, drought acclimation, and non-acclimation, respectively

Moreover, S2 resulted in a significant (p ≤ 0.05) increase in soluble sugar, sucrose, fructose, and glucose contents in the shoot and root tissues of non-acclimated plants from both genotypes. However, the levels of these indicators in drought-acclimated (DA) plants were comparable to those in control (CK) plants (Fig. 3). In leaf tissues, the contents of soluble sugar, sucrose, fructose, and glucose increased by 10%, 8%, 3%, and 23% in DA plants, respectively, and by 31%, 43%, 68%, and 68% in NA plants. Conversely, in root tissue, there was a 27% increase in soluble sugar, 2% in sucrose, 6% in fructose, and 5% in glucose for DA plants, while NA plants exhibited a 77% increase in soluble sugar, 47% in sucrose, 36% in fructose, and 42% in glucose compared to the control (Table 3). Additionally, stress treatment (T) affected both leaf and root soluble sugar, sucrose, fructose, and glucose, whereas genotype (G) influenced only root soluble sugar, sucrose, and glucose content. The interaction between genotype and treatment (G x T) influenced root soluble sugar, sucrose, and glucose content, as revealed by the two-way ANOVA (Table 4).

**Fig. 4:**
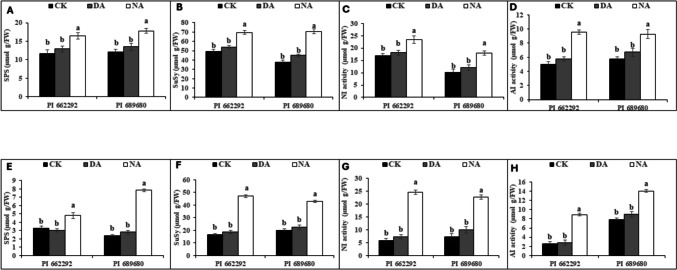
Effect of drought acclimation on the activities of sugar metabolism-related enzymes. (A) sucrose phosphate synthase, (B) sucrose synthase, (C) neutral invertase, and (D) soluble acid invertase activity in the leaves, and (E) sucrose phosphate synthase, (F) sucrose synthase, (G) neutral invertase e, and (H) soluble acid invertase activity in the roots of control (CK), drought-acclimated (DA), and non-acclimated (NA) plants after the second stress (S2) treatment. The data represent the mean (± S.E.) from biological triplicates. Significant differences are indicated by distinct letters on the error bars, with significance set at a probability level of (p ≤ 0.05). CK, DA, NA, and DAT represent control, drought acclimation, non-acclimation, and days after seedling transfer, respectively

Similarly to the changes observed in soluble sugar, sucrose, fructose, and glucose contents, the activities of their associated enzymes; SPS, SuSy, NI, and AI, increased significantly in the leaves and root tissue of millet genotypes following the S2 treatment. The elevation in the levels of these enzymes was more pronounced in NA plants, whereas DA plants of both genotypes exhibited comparable enzymatic activity to that of the control (CK) plants (Fig. 4). For instance, leaf enzymatic activity increased by 9% (SPS), 9% (SuSy), 8% (NI), and 13% (AI) for DA plants, while NA plants showed increases of 29% (SPS), 33% (SuSy), 31% (NI), and 45% (AI). In root tissues, enzymatic activity increased by 3% (SPS), 10% (SuSy), 22% (NI), and 11% (AI) for DA plants, whereas NA plants exhibited increases of 43% (SPS), 62% (SuSy), 74% (NI), and 63% (AI) (Table S3). Additionally, stress treatment (T) affected both leaf and root SPS, SuSy, NI, and AI activities, while genotype (G) influenced leaf NI and AI activities, as well as root SPS and SuSy activity. The interaction between genotype and treatment (G x T) affected only root SPS, SuSy, NI, and AI activities (Table 4).

**Fig. 5:**
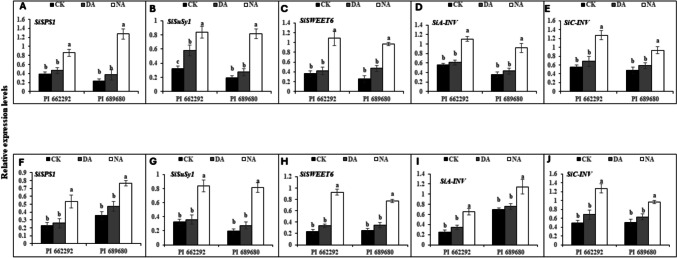
Expression of sugar-related transcripts in the leaves and roots of millet genotypes after subsequent stress (S2) treatment. The relative expression levels of (A) *SiSPS1*, (B) *SiSuSy1*, (C) *SiSWEET6*, (D) *SiA-INV*, and (E) *SiC-INV* in the leaves, and (F) *SiSPS1*, (G) *SiSuSy1*, (H) *SiSWEET6*, (I) *SiA-INV*, and (J) *SiC-INV* in the roots of millet genotypes. The data represent the mean (± S.E.) from biological triplicates. Significant differences are indicated by distinct letters on the error bars, with significance set at a probability level of (p ≤ 0.05). CK represents the control group and DA represents the drought-acclimated, and NA denotes non-acclimated plant groups

In parallel with the contents of soluble sugars (fructose, sucrose, and glucose) and the activities of their associated enzymes (SPS, SuSy, NI, and AI), the expression levels of sugar-metabolism related transcripts; *SiSPS1*, *SiSuSy1*, *SiSWEET6*, *SiA-INV*, and *SiC-INV* were significantly upregulated in both the leaves and root tissues of millet genotypes following S2 treatment. Interestingly, the expression of these transcripts was higher in NA plants, while DA plants exhibited relative expression levels similar to those observed in CK plants (Fig. 5). Specifically, the fold-changed expression of these genes ranged from 1.15-fold (*SiA-INV*) to 1.6-fold (*SiSuSy1*) in DA plants and from 2.12-fold (*SiA-INV*) to fourfold (*SiSPS1*) in NA plants in leaf tissue. In root tissues, the expression level ranged from 1.23-fold (SiSPS1) to 1.43-fold (*SiSWEET6*) in DA plants and from 2.14-fold (*SiA-INV*) to 3.5-fold (*SiSWEET6*) in NA plants of both genotypes. Additionally, stress treatment (T) affected both leaf and root expression levels of *SiSPS1*, *SiSuSy1*, *SiSWEET6*, *SiA-INV*, and *SiC-INV*, while genotype (G) influenced leaf expression levels of *SiSPS1*, *SiSWEET6*, and *SiC-INV*, as well as root expression levels of SiSPS1, SiSuSy1, *SiSWEET6*, *SiA-INV*, and *SiC-INV*. The interaction between genotype and treatment (G x T) affected only the leaf and root expression levels of *SiA-INV*, as shown by the two-way ANOVA results (Table 4).

## Discussion

### Phenotypic response to drought hardening in millet

Prior to undergoing drought treatment (S1), the young seedlings of foxtail millet genotypes 'PI 689680' and 'PI 662292' exhibited synchronized and uniform growth at the fully expanded 3rd leaf stage (Zadoks scale #13) according to the classification of cereal development by Zadoks et al. (1974), depicted in Fig. S1. After 10 d of drought stress (S1), the control plants of PI 689680 displayed better growth, with a greater number of tillers than the control plants of PI 662292 (Fig. S2). Conversely, the drought-stressed plants of PI 689680 exhibited signs of stress, including stunted growth, leaf browning, pale appearance, wilting, and leaf curling, similar to symptoms observed in wheat genotypes under drought stress in our previous study (Amoah et al. 2019). However, no significant differences were observed between the control and drought-stressed plants of PI 662292 after the 10-d drought treatment (Fig. S2). After subsequent stress (S2), both the control (CK) and drought-acclimated (DA) groups of millet genotypes reached the panicle development stage with no observable differences between CK and DA plants (Fig. S3). However, the S2 treatment, implemented to evaluate the influence of hardening (S1) on millet, resulted in heightened drought-related symptoms, including reduced growth, leaf browning, wilting, leaf curling, and the inhibition of panicle initiation in non-acclimated (NA) plants of both genotypes. These symptoms were notably more pronounced in the plants of PI 689680 compared to PI 662292 (Fig. S3). These observations align with previous findings in wheat (Amoah et al. 2019; Khanna-Chopra and Selote 2007; Selote et al. 2004; Selote and Khanna-Chopra 2010), tobacco (Khan et al. 2020, 2021), and millet (Amoah et al. 2023), where the drought-tolerant genotype showed enhanced phenotypic performance compared to the drought-sensitive type. Our data, therefore, highlights the greater tolerance and improved performance of PI 662292, ranking it as the most adaptable to water deficit conditions over PI 689680.

### Drought hardening improved tissue biomass, water content, photosynthetic activity in millet genotypes

**Fig. 6:**
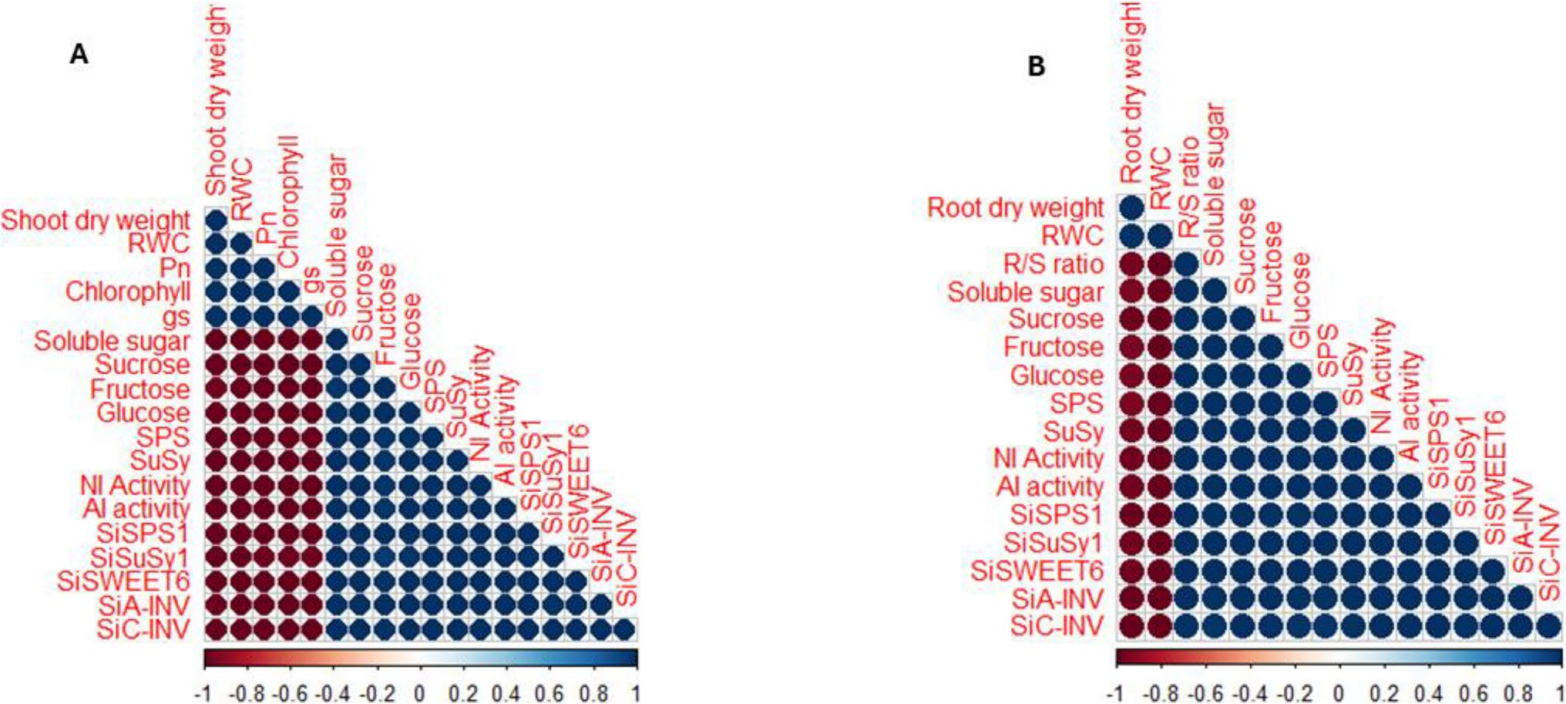
Correlation plot of physiological and molecular indicators studied in the leaves (A) and roots (B) of control (CK) and drought stress (DS) plants of millet genotypes after drought hardening (S1) treatment

The initial drought treatment (S1) significantly (p ≤ 0.05) reduced the shoot and root biomass and water contents, leaf stomatal conductance, net photosynthetic rate, and chlorophyll content (Table 1). The magnitude of change in biomass and relative water content was greater in the shoots than in the roots of millet genotypes (Table 1), which aligns with previous observations in soybean and wheat seedlings under progressive drought conditions (Amoah and Seo 2021; Du et al. 2020b; Selote and Khanna-Chopra 2010). While changes in tissue biomass and water content under drought stress have been well-documented in millet and other plant species (Khan et al. 2020; Khanna-Chopra and Selote 2007; Mude et al. 2023, 2020; Mukami et al. 2019), the dynamics of these changes under drought acclimation conditions, particularly in millet, have not been thoroughly explored. In this study, drought-stressed plants of PI 662292 exhibited higher tissue water content and biomass accumulation than those of PI 689680, consistent with our previous findings (Amoah et al. 2023, 2024). This further demonstrates enhanced physiological functioning, as previously shown by Efeoğlu et al. (2009). As demonstrated by Kaur et al. (2021) and Mahmood et al. (2020), plants store water in their tissues and utilize it during stressful periods to maintain essential physiological processes such as photosynthesis and cell turgidity for an extended duration. In such circumstances, larger tissue biomass and enhanced water storage capacity are key factors that help plants mitigate the drastic effects of drought stress, as shown by Wang et al. (2016) and observed in the drought-stressed plants of PI 662292 in this study (Table 1). Furthermore, a strong positive correlation was found between plant tissue biomass and relative tissue water content, leaf gas exchange parameters, and chlorophyll levels (Fig. 6). This finding aligns with previous observations in soybean (Du et al. 2020b) under progressive drought stress, as well as in wheat (Amoah et al. 2023) and tobacco (Khan et al. 2021) under drought acclimation conditions, suggesting a strong relationship between water availability and photosynthetic activity in millet under drought stress. Additionally, drought-stressed plants of PI 662292 displayed a higher root-to-shoot (R/S) ratio compared to PI 689680 (Table 1). This higher R/S ratio reflects a greater allocation of resources to the roots, as demonstrated by Du et al. (2020a, b) and Liu et al. (2013), leading to increased root growth and enhanced tissue water content (Table 1), thereby conferring greater drought tolerance to PI 662292 compared to PI 689680.

**Fig. 7:**
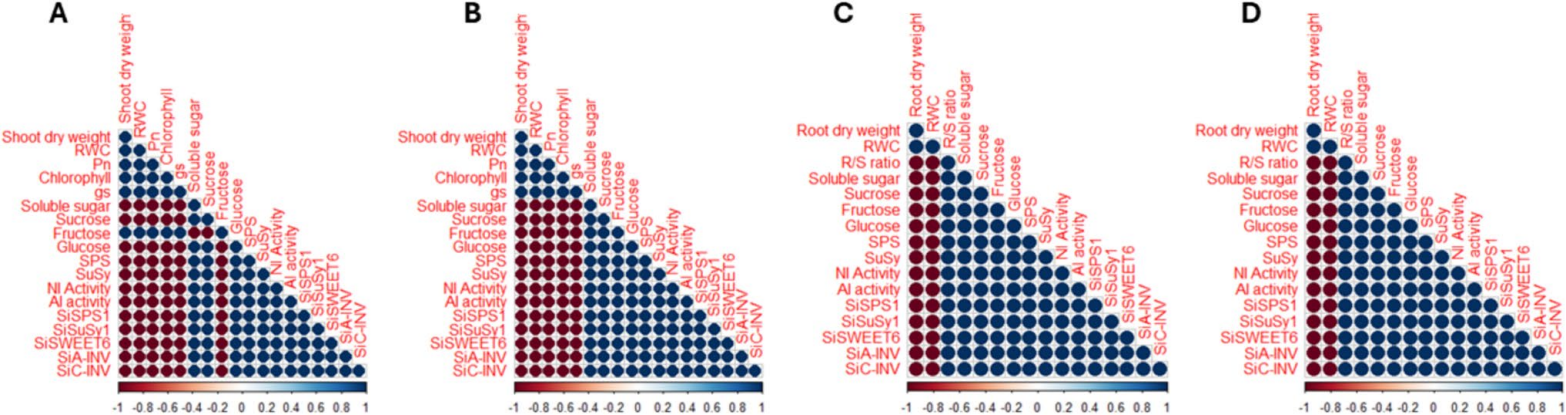
Correlation plot of physiological and molecular indicators studied in leaf (A) of drought acclimated plants, leaf (B) of non-acclimated plants (B), root (C) of drought acclimated plants, and root (D) of non-acclimated plants after drought stress (S2) treatment

Similarly, the second (S2) stress treatment, imposed to determine how millet plants respond to subsequent stress episodes after being hardened, drastically reduced tissue (shoot and root) biomass, relative water content, stomatal conductance, net photosynthetic rate, and chlorophyll content in non-acclimated plants compared to drought-acclimated plants. However, it increased the root-to-shoot (R/S) ratio (Table 3), consistent with previous reports in wheat and soybean (Amoah and Seo 2021; Du et al. 2020b). The relative changes in these physiological indicators in drought-acclimated plants were similar to those observed in the control (CK) plants (Amoah et al. 2019; Khan et al. 2020; Khanna-Chopra and Selote 2007). Interestingly, higher tissue biomass in drought-acclimated (DA) plants during the second stress treatment (S2) correlated with enhanced relative tissue water content and photosynthetic activity (Fig. 7), allowing for lower resource allocation to the roots, as evidenced by a lower root-to-shoot (R/S) ratio (Table 1). This facilitated normal root growth and exploration of a smaller soil volume for water. In contrast, non-acclimated (NA) plants required greater resource allocation to the roots, resulting in a higher R/S ratio (Table 3). This led to increased root growth and the exploration of larger soil volumes for water to ensure plant survival, as shown by Brouwer (1962).

The findings demonstrate that drought hardening induced an acclimation response to the second stress (S2) in drought-acclimated (DA) plants. This response maintained a higher level of photosynthesis, supporting the production of energy and essential compounds to mitigate drought stress. Additionally, a balanced root-to-shoot (R/S) biomass ratio helped ensure sufficient water availability for continued enhanced photosynthesis. This mechanism has been observed in soybean under drought stress (Dong et al. 2023; Du et al. 2020b) and in wheat under drought acclimation conditions (Amoah et al. 2019; Khanna-Chopra and Selote 2007; Selote et al. 2004; Selote and Khanna-Chopra 2010).

### Hardening improved sugar accumulation in drought acclimated plants

The coordinated function of carbon supply and assimilation is crucial for plant growth and development (Muller et al. 2011). Drought hardening (S1) and the second drought stress treatment (S2) reduced the photosynthetic activity of millet genotypes, leading to changes in carbon balance, plant development, and metabolic activities. Both drought-stressed (DS) and non-acclimated (NA) plants exhibited higher levels of stored tissue sugars (Table 1, Fig. 3). During drought stress, soluble sugars such as sucrose, glucose, and fructose can accumulate in plant cells, as shown previously by Dong et al. (2023) and Liang et al. (2021). These sugars increase the cell's osmotic potential, helping to maintain turgor pressure and prevent water loss through osmotic adjustment (Dong et al. 2023; Yang et al. 2023). Sugars also regulate stomatal closure, provide energy for essential processes, scavenge reactive oxygen species (ROS), and protect cellular structures, all of which contribute to drought tolerance (Ho et al. 2001; Loreti et al. 2001). Changes in sugar levels do not follow a static model but fluctuate with genotype and stress conditions, as discovered by Castonguay et al. (1995) and Morsy et al. (2007), aligning with our findings in drought-hardened (DS) and non-acclimated (NA) plants. Sucrose serves as a substrate for cellular respiration or as an osmolyte for maintaining cellular homeostasis. Furthermore, fluctuations in sugar levels under abiotic stress conditions cause alterations in carbon absorption in source-sink dynamics and carbon partitioning (Gupta and Kaur 2005). Increased shoot sugar content has been reported as a drought adaptation mechanism in plants, as it enhances phloem loading, facilitating the flow of sucrose from the leaves to the roots (Du et al. 2020a; Lemoine et al. 2013). Drought hardening (S1) significantly increased the content of soluble sugars, including fructose, sucrose, and glucose, more in drought-stressed plants of PI 689680 compared to PI 662292 (Table 1), consistent with previous findings in wheat and tobacco (Amoah and Seo 2021; Khan et al. 2021). Notably, the levels of these sugars, along with sugar-related enzymes (SPS, SuSy, NI, and AI) and the expression of sugar-related genes (*SiSPS1*, *SiSuSy1*, *SiSWEET6*, *SiC-INV*, and *SiA-INV*), exhibited synchronized enhancement and strong correlations under both drought hardening (in DS) (Table 1, Fig. 2) and the second stress (S2) treatment (in NA) plants (Table 3, Figs. 3–5). These effects were more pronounced in PI 689680 than in PI 662292, suggesting that PI 662292 represents a genotype with enhanced drought tolerance, as shown previously (Amoah et al. 2023; Dong et al. 2023).

Furthermore, after the S2 treatment, drought-acclimated (DA) plants showed tissue soluble sugar content, specifically sucrose and fructose, comparable to control levels (Fig. 3). Normally, plants increase total sugar accumulation as part of their drought adaptation mechanisms, which assists in phloem loading and facilitates sucrose transport from leaves to roots (Du et al. 2020a; Lemoine et al. 2013), as observed in non-acclimated (NA) plants (Fig. 3), akin to observations in non-acclimated (NA) plants (Fig. 3). However, the increased tissue total sugars in DA plants suggest a reduced osmotic potential that facilitated water conservation, thereby aiding adaptation to subsequent stress conditions. Total sugars exhibited a correlation with root-to-shoot biomass ratios (Fig. 7), indicating altered energy and resource allocation within the plant. This allocation pattern corresponded with enhanced stomatal activity, osmotic adjustment, and improved growth following the S2 treatment (Table 3). Moreover, DA plants exhibited significantly lower activities of SPS, SuSy, AI, and NI enzymes compared to NA plants, where enzyme activities were relatively higher (Figs. 4). These enzyme activities correlated with levels of total sugars, including soluble sugars, sucrose, and fructose, as well as with the expression of their respective genes (*SiSPS1*, *SiSuSy*, *SiSWEET6*, *SiA-INV*, and *SiC-INV*) (Fig. 5). Interestingly, while the activities of these enzymes and the expression of their related transcripts have been studied in various species under drought stress conditions (Amoah et al. 2023; Du et al. 2020a, 2020b; Ghouili et al. 2021), information specific to their role during drought acclimation is lacking. These findings suggest that acclimation induced cellular reprogramming or carbon remobilization in DA plants. This reprogramming aided in preventing excessive water loss through enhanced osmotic adjustment, conserving energy, mitigating the risk of osmotic stress and cell damage, and promoting resource allocation toward drought tolerance mechanisms, such as root growth and the synthesis of related genes, following the S2 treatment (Hlahla et al. 2022; Khan et al. 2019; Wang et al. 2019).

## Conclusion

**Fig. 8:**
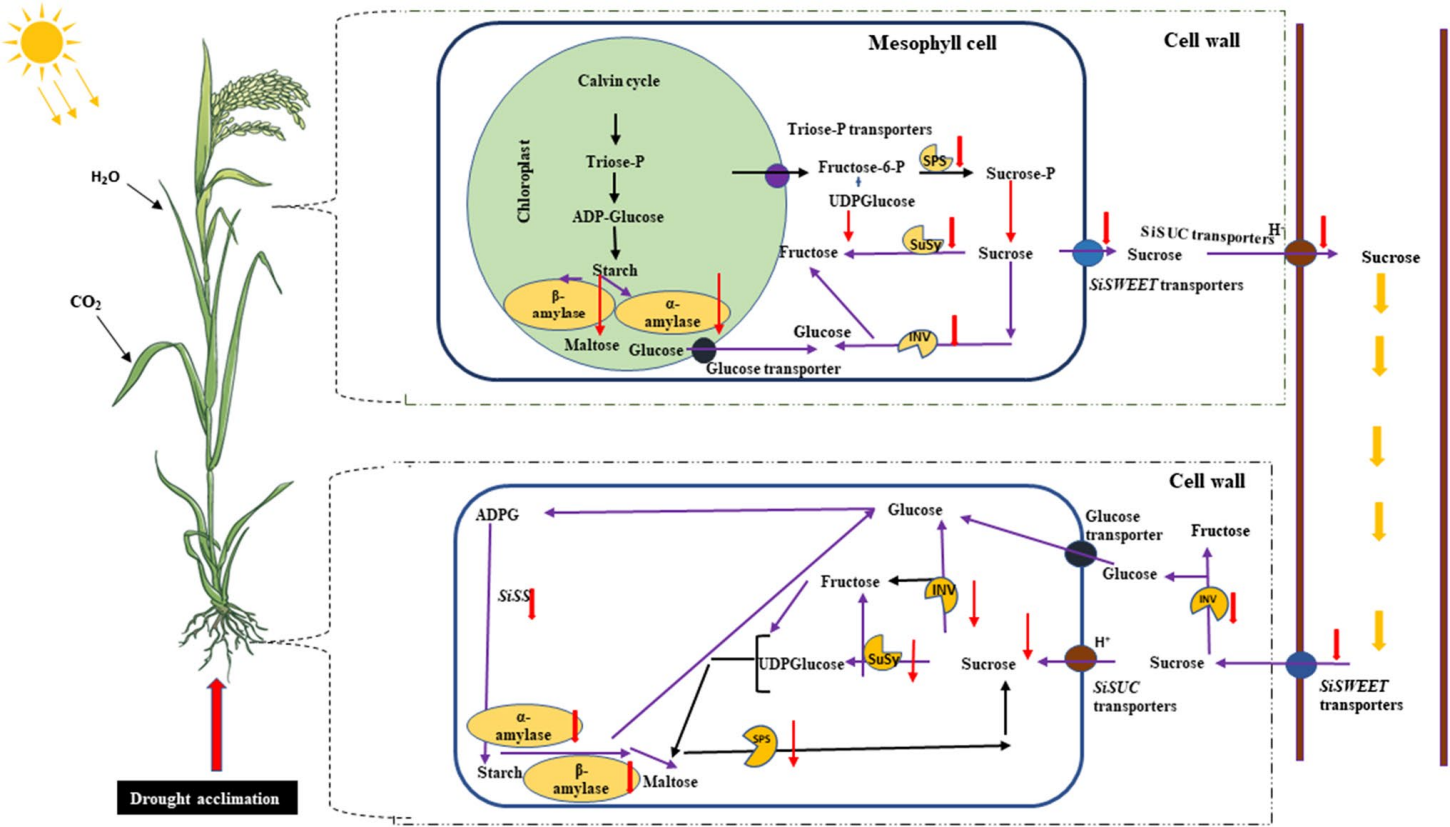
A model of sugar metabolism in drought-acclimated (DA) millet seedlings. Drought hardening (acclimation) causes a sugar-mediated tandem response, which enhances drought tolerance in millets. Drought acclimation altered the expression levels of key regulatory metabolic genes and the activities of sugar metabolism enzymes, modulating sugar accumulation and activating the transcription of sugar transporters to regulate sugar allocation in response to drought stress. Down-regulated items are indicated by downward red arrows

The findings show that subjecting millet plants to a hardening process triggers an acclimation response promoting growth, and regulating sugar allocation, metabolism, and transport in millet under drought stress condition (Fig. 8). Drought acclimated (DA) retained a kind of "memory" from the initial stress (S1), which allowed them to respond more quickly and effectively to subsequent stress (S2) compared to non-acclimated (NA) plants. The hardening process caused an increased accumulation of total sugars in millet plants, particularly in their shoots rather than their roots. This accumulation helped mitigate cellular damage caused by osmotic stress, ensuring that solute concentrations within plant cells were maintained to preserve turgor pressure and cell hydration. This process was aided by enzymes such as sucrose phosphate synthase (SPS), sucrose synthase (SuSy), alkaline invertase (AI), and neutral (NI), which contribute to osmotic adjustment. The results also suggest that hardening plays a role in protecting millet plants from cellular damage through three main mechanisms: first, it enhances osmotic adjustment, leading to lower total sugar accumulation in the roots and reduced translocation to the shoots; second, it reduces starch accumulation while increasing photosynthetic and stomatal activity, chlorophyll content, and water content, all while maintaining a lower root-to-shoot biomass ratio; and third, it leads to the lower regulation of genes related to sugar and starch, which aligns well with the activity of the corresponding enzymes, thus safeguarding vital metabolic processes like photosynthesis and genetic materials such as DNA. However, even though there is some understanding of drought tolerance mechanisms driven by phytohormone applications under drought stress, the study underscores the need for further exploration of the molecular mechanisms that underlie drought acclimation in plants. Such research could offer a more comprehensive understanding of how plants develop resilience to drought, which is crucial for enhancing their ability to withstand drought, maintain high millet yields, and contribute to greater food security in the future. Additionally, the study will offer valuable insights for plant breeders regarding the selection of drought-tolerant millet genotypes in crop improvement programs.

## Supplementary information

Below is the link to the electronic supplementary material.

Supplementary file1 (DOCX 1775 KB)

Supplementary file2 (DOCX 30 KB)

## Data Availability

Data is available upon request from the corresponding author.
