# Supplementary material for: Effect of drought acclimation on sugar metabolism in millet
Source: Protoplasma. 2024 Aug 5;262(1):35–49. doi: 10.1007/s00709-024-01976-5 (PMC11698784; doi:10.1007/s00709-024-01976-5)
Supplement: Supplementary file 1 — Supplementary file1 (DOCX 1775 KB) [file 709_2024_1976_MOESM1_ESM.docx]

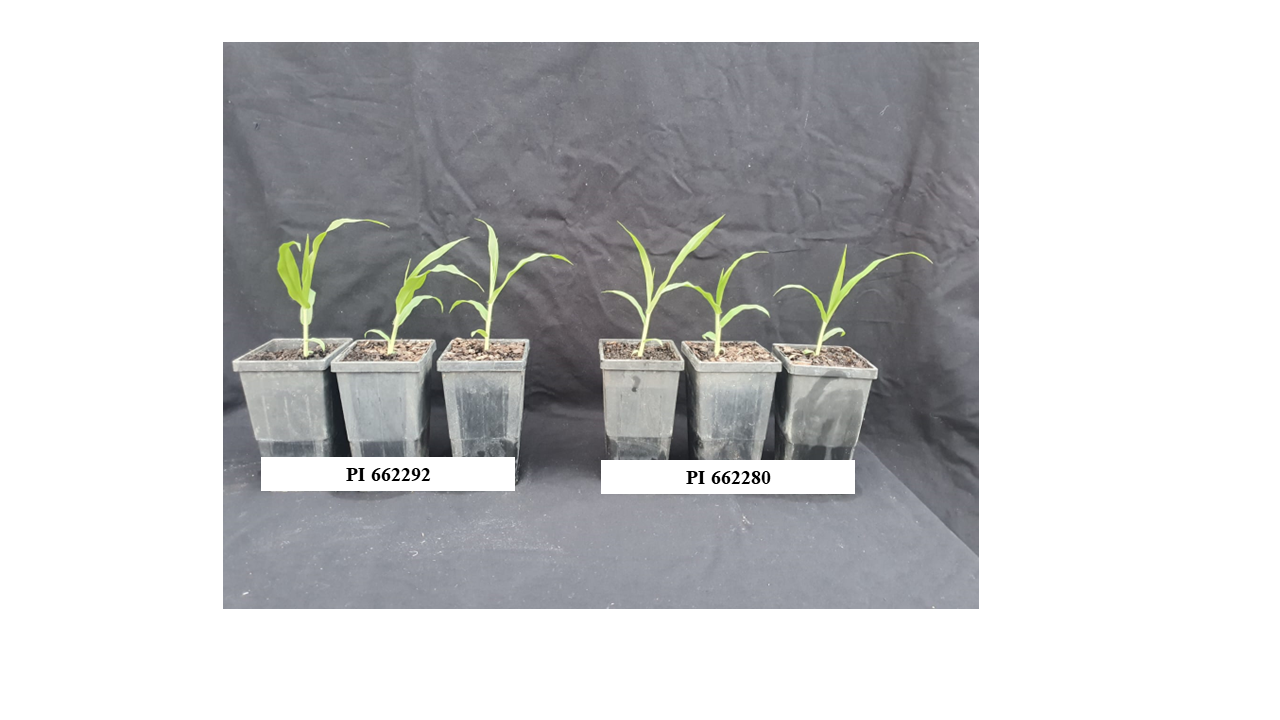


**Fig. S1**: Millet genotypes at fully expanded 3rd leaf stage, according to Zadok’s scale #21 and prior to drought hardening (S1) treatment.


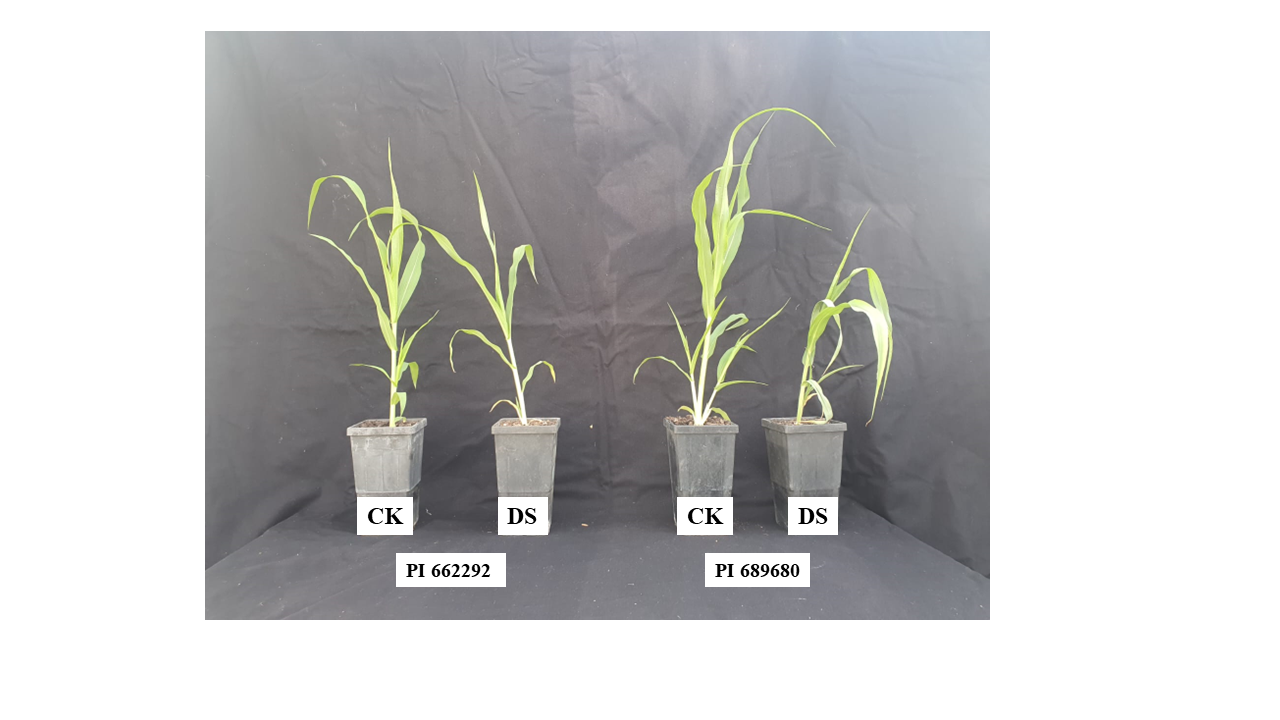


**Fig. S2:** Seedlings of millet genotypes after drought hardening treatment. CK and DS represents control and drought stress, respectively.


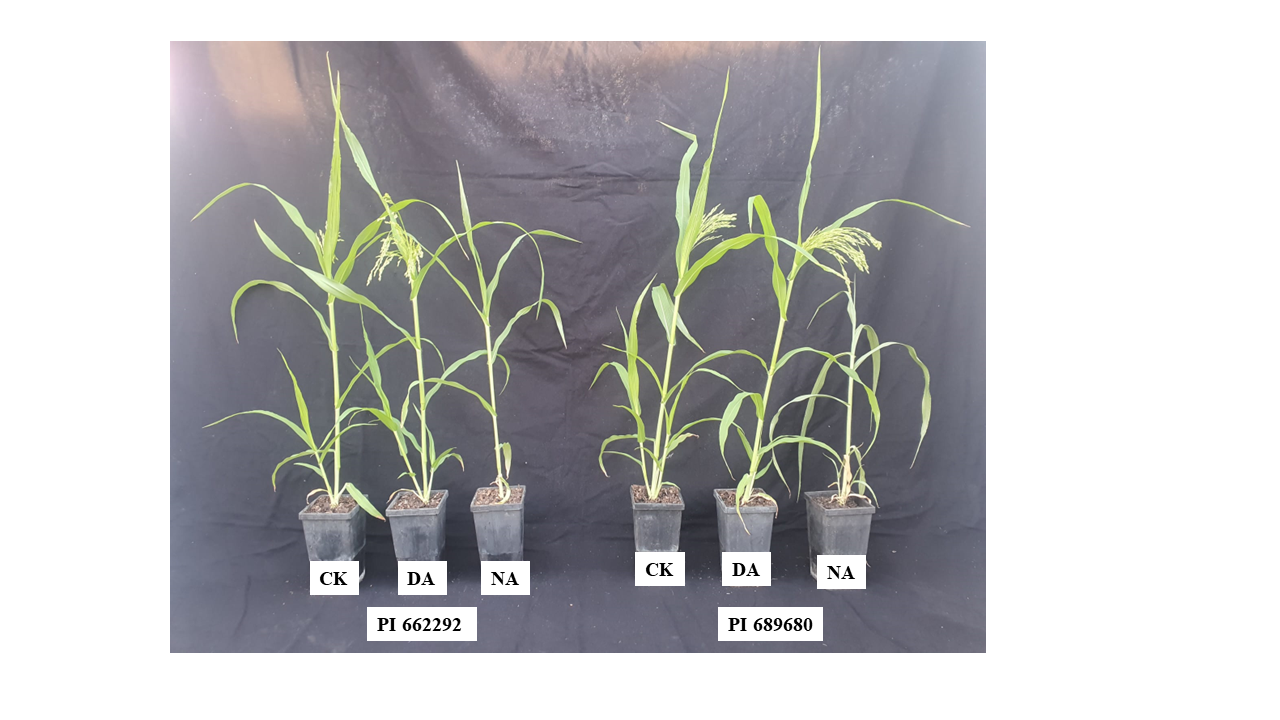


**Fig. S3:** Seedlings of millet genotypes after drought hardening treatment. CK, control; DA, drought control acclimation and, NA, non-acclimation, respectively. CK, DA and NA represents control and drought acclimation, and non-acclimation, respectively.
