# Supplementary material for: Effect of drought acclimation on sugar metabolism in millet
Source: Protoplasma. 2024 Aug 5;262(1):35–49. doi: 10.1007/s00709-024-01976-5 (PMC11698784; doi:10.1007/s00709-024-01976-5)
Supplement: Supplementary file 2 — Supplementary file2 (DOCX 30 KB) [file 709_2024_1976_MOESM2_ESM.docx]

**Table S1: List of primers used for the quantitative polymerase chain reaction (qPCR) analysis.**

| Gene ID | Forward primer (5’ – 3’) | Reverse primer (3’ – 5’) | Reference |
| --- | --- | --- | --- |
| *SiSPS1*  *SiSuSy1*  *SiSWEET6*  *SiC-INV*  *SiA-INV*  *SiTublin1*  *SiActin*  *SiUBQ1* | GGTTTGGCTACAGGAGTCCT  GAAGGTTTCTGCTGCTAGGC  CGGTGCCACATCATGTACTG  GGAGGGCCAGCTGAGATTAT  ACCCCACAAAGTCGTCTCTT  ACTGAGCCACTGACAATCGA  TCCTCAGAGACAATGCACGT  GCAGGCCATTATGTCGTTCC | CAAATCCATGGAAACCGGCA  TCCAAAGCCCATGACAAAGC  GCATCTCTTCAGCATCGGTG  TTCCAGAAACCGTAGACCCC  CATCCGCCTCTCCATTGTTG  TACGACATTGACGCTTGCAG  CTTCCAAGCCTCCTTCATGC  GCTCTTCATGAAATCGGCGT | Du et. al (2019), Du et. al. (2020) and Dong et. al (2023)  Du et. al (2019), Du et. al. (2020) and Dong et. al (2023)  Du et. al (2019), Du et. al. (2020) and Dong et. al (2023)  Du et. al (2019), Du et. al. (2020) and Dong et. al (2023)  Du et. al (2019), Du et. al. (2020) and Dong et. al (2023)  Du et. al (2019)  Du et. al (2019)  Dong et. al (2023) |

**Table S2: Change (%) in physiological, biochemical and transcript regulation under drought treatment (S1) condition.**

| Traits/(plant) | Leaf | | Root | |
| --- | --- | --- | --- | --- |
|  | **PI 662292** | **PI 689680** | **PI 662292** | **PI 689680** |
|  | **DS** | **DS** | **DS** | **DS** |
| Dry weight  RWC  Pn  Chlorophyll  gs  R/S ratio  Soluble sugar  Sucrose  Fructose  Glucose  SPS  SuSy  NI  AI  SiSPS1  SiSuSy1  SiSWEET6  SiA-INV  SiC-INV | -22.46  -10.96  -13.42  -7.40  -13.42  2.78  30.41  41.79  31.55  30.00  65.00  41.79  52.35  46.41  1.67  5.61  4.08  3.18  1.54 | -34.03  -26.85  -59.77  -52.54  -59.77  34.57  40.77  41.98  30.32  60.00  80.00  23.98  30.80  40.76  4.88  1.80  3.00  4.03  12.66 | -19.19  -20.86  N/A  N/A  N/A  2.78  22.18  19.59  15.69  15.79  20.16  27.12  23.75  25.43  2.22  1.83  3.33  3.28  3.34 | -68.21  -52.22  N/A  N/A  N/A  34.57  36.71  40.74  42.20  60.82  30.32  74.65  36.88  40.97  2.38  3.88  3.20  2.52  4.19 |

DS; drought stress RWC, relative water content; Pn, Net photosynthetic rate; gs, stomatal conductance; R-S ratio, root/shoot ratio; SPS//*SPS1*, sucrose phosphate synthase; SuSy//SuSy1, sucrose synthase; INV, invertase; NI, neutral invertase; and AI, acid invertase. Negative (-) and positive (+) values indicate a percentage (%) decrease and increase, respectively.

**Table S3****: Change (%) in physiological, biochemical and transcript regulation under drought treatment condition.**

| Plant / (traits) | Leaf | | | |  |  |  |  | | Root | |  |  |
| --- | --- | --- | --- | --- | --- | --- | --- | --- | --- | --- | --- | --- | --- |
|  | **PI 662292** | | **PI 689680** | | **Average** |  |  | **PI 662292** | | **PI 689680** | |  | **Average** |
|  | **DA** | **NA** | **DA** | **NA** | **DA** | **NA** |  | **DA** | **NA** | **DA** | **NA** | **DA** | **NA** |
| Dry weight  RWC  Pn  Chlorophyll  gs  R-S ratio  Soluble sugar  Sucrose  Fructose  Glucose  SPS  SuSy  NI Activity  AI activity | -9.30  -8.58  -7.94  -4.91  -4.73  N/A  9.30  8.92  6.67  31.18  9.30  7.95  7.35  13.37 | -80.77  -68.07  -110.03  -105.31  -104.85  N/A  31.67  47.93  67.44  69.35  28.91  29.00  28.31  47.90 | -16.92  -3.59  -0.44  -2.32  -2.23  N/A  10.29  7.39  13.16  4.01  10.29  16.36  14.96  14.14 | -68.11  -30.86  -56.00  -50.91  -50.66  N/A  32.11  31.70  71.52  65.90  32.11  46.74  43.04  37.77 | -11.24  -7.23  -6.01  -4.26  -4.10  N/A  9.55  8.47  2.82  23.17  9.55  9.86  8.87  13.58 | -77.33  -57.08  -94.64  -89.74  -89.33  N/A  31.78  43.70  68.40  68.43  29.74  33.47  31.50  45.40 |  | -17.78  -3.97  N/A  N/A  N/A  31.54  0.63  5.97  5.09  1.70  8.29  9.71  20.00  10.71 | -59.00  -39.72  N/A  N/A  N/A  79.06  38.74  37.15  34.02  71.58  31.94  64.45  76.22  71.91 | -13.79  -8.41  N/A  N/A  N/A  15.48  5.51  8.33  4.70  17.81  14.29  11.47  27.24  12.27 | -57.14  -46.44  N/A  N/A  N/A  71.88  69.23  30.92  62.37  75.52  69.36  53.41  67.79  43.94 | -16.75  -5.06  N/A  N/A  N/A  27.01  1.55  6.62  5.01  5.42  2.85  10.20  22.09  11.31 | -58.52  -41.36  N/A  N/A  N/A  77.11  46.85  35.54  41.70  72.48  43.54  61.81  74.20  63.35 |
|  |  |  |  |  |  |  |  |  |  |  |  |  |  |
| Plant/ (trait) | **Leaf** | |  | |  |  |  | **Root** | |  | |  |  |
|  | **PI 662292** | | **PI 689680** | | **Average** |  |  | **PI 662292** | | **PI 689680** | | **Average** |  |
|  | **DA** | **NA** | **DA** | **NA** | **DA** | **NA** |  | **DA** | **NA** | **DA** | **NA** | **DA** | **NA** |
| *SiSPS1*  *SiSuSy1*  *SiSWEET6*  *SiA-INV*  *SiC-INV* | 1.21  1.79  1.16  1.10  1.24 | 2.24  2.59  3.01  1.98  2.32 | 1.61  1.41  1.87  1.20  1.22 | 5.57  4.17  3.81  2.56  1.94 | 1.41  1.60  1.52  1.15  1.23 | 3.91  3.38  3.41  2.27  2.13 |  | 1.12  1.11  1.46  1.40  1.38 | 2.31  2.59  4.04  2.64  2.57 | 1.34  1.41  1.40  1.09  1.24 | 2.17  4.17  3.12  1.64  1.90 | 1.23  1.26  1.43  1.25  1.31 | 2.24  3.38  3.58  2.14  2.24 |

DA, drought acclimation; NA, non-acclimation; RWC, relative water content; Pn, Net photosynthetic rate; gs, stomatal conductance; R-S ratio, root/shoot ratio; SPS//*SPS1*, sucrose phosphate synthase; SuSy//SuSy1, sucrose synthase; INV, invertase; NI, neutral invertase; and AI, acid invertase. Negative (-) and positive (+) values indicate a percentage (%) decrease and increase, respectively.
